# Trends in the susceptibility of U.S. *Acinetobacter baumannii-calcoaceticus* species complex and *Stenotrophomonas maltophilia* isolates to minocycline, 2014–2021

**DOI:** 10.1128/spectrum.01981-23

**Published:** 2023-11-03

**Authors:** Michael A. Pfaller, Dee Shortridge, Cecilia G. Carvalhaes, Mariana Castanheira

**Affiliations:** 1 University of Iowa, Iowa City, Iowa, USA; 2 JMI Laboratories, North Liberty, Iowa, USA; Michigan State University, East Lansing, Michigan, USA

**Keywords:** *Acinetobacter*, *Stenotrophomonas*, minocycline, Surveillance

## Abstract

*Acinetobacter baumannii*-*calcoaceticus* species complex (ACB) and *Stenotrophomonas maltophilia* (SM) are opportunistic, non-fermentative organisms that can cause serious hospital-acquired infections in immunocompromised patients. These pathogens are inherently resistant to several common drug classes and often acquire other resistance mechanisms, making them difficult to treat. In this study, we analyzed the susceptibility of 1,029 contemporary ACB and 1,522 SM isolates to minocycline (MIN) and levofloxacin (LEV) as well as meropenem (MER) for ACB and trimethoprim-sulfamethoxazole (TMP/SMX) for SM using the CLSI broth microdilution method. Isolates were collected as a part of the SENTRY Antimicrobial Surveillance Program from 2014 to 2021. Pneumonia in hospitalized patients was the most common infection from which ACB (57.0%) and SM (73.9%) were isolated. MIN had the highest *in vitro* activity for ACB (86.2%) and SM (99.5%). The activity of ACB and SM to all three agents varied over the period studied. MIN activity to ACB decreased in 2020 (80.6%) but rebounded in 2021 (86.2%). LEV and MER showed an overall trend of increasing susceptibility for ACB, with slightly lower activity in 2020–2021. MIN and TMP/SMX (>98.3% and >93.7%, respectively) activities were stable against SM isolates. LEV activity decreased from 84.3% (2015) to 69.2% (2018). The activity of MIN remained stable and higher than other agents tested for both ACB and SM, pathogens that have limited therapeutic alternatives. These *in vitro* data suggest that MIN is a useful treatment option for infections caused by ACB or SM.

**IMPORTANCE:**

*Acinetobacter baumannii-calcoaceticus* species complex and *Stenotrophomonas maltophilia* are opportunistic, non-fermentative Gram-negative organisms that can cause serious hospital-acquired infections in immunocompromised patients. These pathogens are inherently resistant to several common drug classes and often acquire other resistance mechanisms, making them difficult to treat. In this study, we analyzed the trends of susceptibility of over 2,500 U.S. bacterial isolates collected from hospitalized patients over an 8-year period to minocycline, which is used to treat infections caused by these pathogens. These *in vitro* data suggest that minocycline is a useful treatment option for infections caused by *Acinetobacter baumannii-calcoaceticus* species complex or *Stenotrophomonas maltophilia*.

## INTRODUCTION

*Acinetobacter baumannii-calcoaceticus* species complex (ACB) and *Stenotrophomonas maltophilia* (SM) are opportunistic, non-fermentative Gram-negative bacilli (GNB) that can cause serious hospital-acquired infections in immunocompromised patients (1). The ACB includes the difficult-to-distinguish species *Acinetobacter baumannii*, *Acinetobacter nosocomialis*, and *Acinetobacter pittii*, as well as the environmental species *Acinetobacter calcoaceticus* (2). These species colonize the skin and respiratory tract of healthy individuals and rarely are the cause of community-onset infections. However, in nosocomial settings, especially in intensive care units, the frequency of infections due to ACB has increased in recent years (1 – 3). The crude mortality rate associated with infections involving ACB is estimated at 26.0% to 61.6% and may often be ascribed to either delayed or inadequate empirical therapy (4 – 6). The selection of an optimal empirical antimicrobial that covers ACB is complicated by the fact that organisms in this species complexes have acquired genes encoding resistance to virtually all agents capable of treating GNB infections, including fluoroquinolones, aminoglycosides, and cephalosporins (1 – 3). Carbapenems are usually the drug of choice for ACB infections; however, the production of carbapenemases, alteration of porin proteins, and hyperexpression of drug efflux systems have significantly impacted the efficacy of this class of agents (2 – 4, 6). The multidrug-resistant (resistant to ≥3 classes of agents) nature of ACB has led to the use of the toxic polymyxin class of antimicrobials as agents of choice for treating such infections (2, 3). The effective use of polymyxins to treat infections due to ACB is limited by their toxicity and the development of resistance to this class of agents in many geographic regions (1, 7, 8).

SM occupies a similar niche to ACB, as it causes nosocomial pneumonia in mechanically ventilated individuals as well as bloodstream infections (BSI) in neutropenic patients (1, 9, 10). Treatment of infections due to SM is limited by the intrinsic resistance of SM to most antimicrobial agents, including penicillin, cephalosporins, carbapenems, and aminoglycosides (1, 11, 12). Trimethoprim-sulfamethoxazole (TMP/SMX) is considered the drug of choice for treating infections due to SM, although TMP/SMX resistance has emerged in SM isolates worldwide (1, 13, 14). As with ACB, delayed and/or inappropriate empirical therapy for SM infections is associated with higher mortality rates (15).

The tetracycline class of antibacterial agents has been employed as broad-spectrum activity against Gram-negative and Gram-positive bacteria) agents since the 1940s (16, 17). Minocycline is a second-generation enhanced tetracycline with improved activity against SM and ACB compared to older members of the class (16, 18, 19). Minocycline is one of the few antibacterial agents that has been approved by the U.S. Food and Drug Administration (FDA) for treatment of infections due to ACB (20). The intravenous formulation of minocycline was granted the status of a Qualified Infectious Disease Product by the U.S. FDA for the treatment of infections due to SM and *Burkholderia cepacia* in patients with cystic fibrosis and chronic granulomatous disease (20).

The aim of the present study is to examine the *in vitro* activity of minocycline and comparator agents against a collection of contemporary U.S. ACB and SM isolates. These isolates were collected from 2014 to 2021 from 35 medical centers during the SENTRY Antimicrobial Surveillance Program (1).

## RESULTS

A total of 1,029 ACB isolates and 1,522 SM isolates from the SENTRY Antimicrobial Surveillance Program collection were evaluated, representing 35 U.S. medical centers from 2014 to 2021 (Table 1). The isolates were collected from clinical specimens including hospitalized patients (PHP) (587 isolates, 57.1% ACB; 1,124 isolates, 73.9% SM), skin and skin structure (SSSI) (216 isolates, 21.0% ACB; 112 isolates, 7.4% SM), BSI (137 isolates, 13.3% ACB; 196 isolates, 12.9% SM), intra-abdominal (IAI) (32 isolates, 3.1% ACB; 57 isolates, 3.8% SM), urinary tract (UTI) (55 isolates, 5.3% ACB; 31 isolates, 2.0% SM), and other infections (1 isolate, 0.1% ACB; 2 isolates, 0.1% SM). Table 1 shows the proportion of isolates of ACB and SM that were susceptible to minocycline, levofloxacin, meropenem (ACB), and TMP/SMX (SM) for each study year and all years combined. Tables 2 and 3 show the MIC distributions of ACB and SM to minocycline and comparators, respectively.

**TABLE 1:** Susceptibilities of *Stenotrophomonas maltophilia* and *Acinetobacter baumannii-calcoaceticus* species complex to minocycline and its comparators, 2014–2021

Organism	% of susceptibility								
	2014^*a*^	2015	2016	2017	2018	2019	2020	2021	Total
ACB^*b*^ (n)	(100)	(155)	(176)	(103)	(151)	(137)	(98)	(109)	(1029)
MIN	88.0%	86.5%	85.8%	81.6%	92.1%	86.1%	80.6%	86.2%	86.2%
LEV	50.0%	45.8%	56.8%	59.2%	66.9%	68.6%	67.3%	61.5%	59.3%
MER	52.0%	51.0%	55.1%	66.0%	66.9%	71.5%	67.3%	66.1%	61.5%
SM (*n*)	(190)	(223)	(209)	(196)	(172)	(224)	(151)	(157)	(1522)
MIN	99.5%	100.0%	99.5%	100.0%	98.3%	99.1%	100.0%	99.4%	99.5%
LEV	71.8%	84.3%	81.8%	79.1%	69.2%	72.8%	82.0%	82.8%	77.9%
TMP/SMX	95.7%	93.7%	93.8%	94.9%	95.3%	94.6%	99.3%	97.5%	95.4%

^*a*^ CLSI (M100, 2023).

^*b*^ ACB, *Acinetobacter baumannii-calcoaceticus* species complex; MIN, minocycline; LEV, levofloxacin; MER, meropenem; SM, *Stenotrophomonas maltophilia*, TMP/SMX, trimethoprim-sulfamethoxazole.

**TABLE 2:** MIC distributions of *Acinetobacter baumannii-calcoaceticus* complex, all isolates, and meropenem-resistant isolates

Antimicrobial agent	MIC (mg/L)											MIC_50_	MIC_90_
	0.06	0.12	0.25	0.5	1	2	4	8	16	32	>^*a*^		
All (*n* = 1,029)													
Minocycline	149	245	187	75	84	99	**48^*b*^**	51			91	0.25	8
	14.5%	38.3%	56.5%	63.8%	71.9%	81.5%	**86.2%**	91.2%			100.0%		
Levofloxacin		365	176	54	12	**3**	17				402	0.25	>4
		35.5%	52.6%	57.8%	59.0%	**59.3%**	60.9%				100.0%		
Meropenem	4	44	271	193	83	**38**	20	17	51	83	225	1	>32
	0.4%	4.7%	31.0%	49.8%	57.8%	**61.5%**	63.5%	65.1%	70.1%	78.1%	100.0%		
Meropenem-R (*n* = 376)													
Minocycline	2	7	19	38	64	80	**39**	42			85	2	>8
	0.5%	2.4%	7.4%	17.6%	34.6%	55.9%	**66.2%**	77.4%			100.0%		
Levofloxacin			0	1	2	**0**	10				363	>4	>4
			0.0%	0.3%	0.8%	**0.8%**	3.5%				100.0%		
Meropenem						**0**	0	17	51	83	225	>32	>32
						**0.0%**	0.0%	4.5%	18.1%	40.2%	100.0%		

^*a*^ Greater than the highest dilution tested.

^*b*^ Susceptible breakpoint in bold font.

**TABLE 3:** MIC distributions of *S. maltophilia*, all isolates, trimethoprim-sulfamethoxazole resistant isolates, and levofloxacin non-susceptible isolates

Antimicrobial agent	MIC (mg/L)									MIC_50_	MIC_90_
	0.06	0.12	0.25	0.5	1	2	4	8	>^*a*^		
All (*n* = 1,522)											
Minocycline	3	70	367	578	305	143	**48^*b*^**	5	3	0.5	2
	0.2%	4.8%	28.9%	66.9%	86.9%	96.3%	**99.5%**	99.8%	100.0%		
Levofloxacin		10	50	281	566	**279**	142		193	1	>4
		0.7%	3.9%	22.4%	59.6%	**78.0%**	87.3%		100.0%		
Trimethoprim-sulfamethoxazole				1356	54	**41**	25		45	≤0.5	1
				89.2%	92.7%	**95.4%**	97.0%		100.0%		
Trimethoprim-sulfamethoxazole -R (*n* = 70)											
Minocycline	0	1	6	13	18	16	**9**	4	3	1	4
	0.0%	1.4%	10.0%	28.6%	54.3%	77.1%	**90.0%**	95.7%	100.0%		
Levofloxacin		0	1	2	8	**7**	11		41	>4	>4
		0.0%	1.4%	4.3%	15.7%	**25.7%**	41.4%		100.0%		
Trimethoprim-sulfamethoxazole						**0**	25		45	>4	>4
						**0.0%**	35.7%		100.0%		
Levofloxacin-NS (*n* = 335)											
Minocycline		0	10	45	111	118	**43**	5	3	2	4
		0.0%	3.0%	16.4%	49.6%	84.8%	**97.6%**	99.1%	100.0%		
Levofloxacin						**0**	142		193	>4	>4
						**0.0%**	42.4%		100.0%		
Trimethoprim-sulfamethoxazole				218	37	**28**	18		34	≤0.5	>4
				65.1%	76.1%	**84.5%**	89.9%		100.0%		

^*a*^ Greater than the highest dilution tested.

^*b*^ Susceptible breakpoint in bold font.

### Activity against the ACB

Minocycline was the most active agent against ACB, exhibiting MIC_50/90_ values at 0.25/8 mg/L and 86.2% susceptibility (Tables 1 and 2). The activity for levofloxacin (MIC_50/90_, 0.25/>4 mg/L, 59.3% susceptible) and meropenem (MIC_50/90_, 1/>32 mg/L, 61.5%) was considerably lower than that for minocycline (Tables 1 and 3). The activity of antimicrobial agents against ACB varied over the 8-year duration of the study. The susceptibility of ACB to minocycline peaked at 92.1% in 2018 and decreased to 80.6% in 2020 and rebounded to 86.2% in 2021. Levofloxacin and meropenem showed an overall trend of increasing susceptibility, with the lowest percentage of susceptibility for both agents observed in 2015 (45.8% for levofloxacin and 51.0% for meropenem) and the highest activity for both in 2019 (68.6% for levofloxacin and 71.5% for meropenem) with a subsequent decline to 61.5% (levofloxacin) and 66.1% (meropenem) in 2021. There were 376 isolates of ACB (36.5%) that were resistant to meropenem, 66.2% of which were susceptible to minocycline and 0.8% were susceptible to levofloxacin (Table 2).

### Activity against SM

The highest susceptibility rate against SM was that of minocycline (99.5% susceptible), with MIC_50/90_ values at 0.5/2 mg/L (Tables 1 and 3). TMP/SMX activity was 95.4% susceptible with MIC_50/90_ values of 0.5/1 mg/L (Tables 1 and 3). Levofloxacin showed moderate activity (77.9% susceptible; MIC_50/90_, 1/>4 mg/L). Both minocycline and TMP/SMX maintained a high level of activity over time with no clear trend of increasing or decreasing activity. There was no trend of increased or decreased activity for levofloxacin over time. The activity of minocycline was maintained for SM isolates that were resistant to TMP/SMX (90.0% susceptible; MIC_50/90_, 1/4 mg/L) or non-susceptible to levofloxacin (97.6% susceptible; MIC_50/90_, 2/4 mg/L) (Table 3).

## DISCUSSION

These data confirm and extend our previous observations regarding the *in vitro* activity of minocycline against isolates of ACB and SM (18, 19). Minocycline was active against more than 86% of clinical isolates of ACB (86.2%) and SM (99.5%). This level of activity was stable for both organisms over the course of the study (Table 1).

The minocycline breakpoints against ACB have been questioned since a modern PK/PD evaluation was not performed for tetracyclines and these agents against ACBs (21). Recently, Lepak et al. demonstrated that when using these updated evaluations, the susceptible minocycline breakpoints for ACB would be≤0.5 mg/L or ≤1 mg/L for standard high-dose minocycline regimens of 200 mg per day or 200 mg q12h, respectively (22). These breakpoints have not been discussed by regulatory agencies (U.S. FDA or EMA) or standard development organizations (CLSI and EUCAST) nor have formal well-designed clinical trials been performed to correlate minocycline MIC values with clinical outcomes; however, they would produce a susceptibility rate of 63.8% or 71.9% for the ACB isolates analyzed in this study at ≤0.5 mg/L or ≤1 mg/L, respectively.

Meropenem is generally considered to be the drug of choice for the treatment of infection due to ACB (20). Although the proportion of ACB susceptible to meropenem showed a slight trend toward increasing over time, the overall resistance rate was high at 36.5%, posing a problem for the empirical use of this agent (Table 1). Whereas minocycline was active against 66.2% of the meropenem-resistant isolates, levofloxacin was only active against 0.8% of these isolates. Minocycline may be preferred over colistin for the treatment of mild infections due to carbapenem-resistant *Acinetobacter baumannii* (CRAB) due to a more favorable toxicity profile (20). Combination therapy with minocycline plus one additional active agent is recommended for the treatment of moderate to severe CRAB infections given the limited clinical data to support the use of monotherapy (20). Also, this IDSA Guidance document suggests using maximum doses of minocycline, such as 200 mg IV q12h, as monotherapy or combination therapy. Clinical trials designed to determine the utility of combination therapy for CRAB have largely been inconclusive (20). Despite the lack of convincing data favoring combination therapy, combination therapy still is recommended for moderate to severe infection due to the lack of data supporting the efficacy of monotherapy in this situation and to the concern that, given the nature of the organism, resistance may develop to a single agent during the course of therapy (1, 20).

Either TMP/SMX or minocycline is considered the preferred agent for the treatment of mild infection due to SM (20). Although TMP/SMX has traditionally served as the agent of first choice for the treatment of SM infections, clinical data have shown no difference in outcomes for patients treated with TMP/SMX versus minocycline (12). The use of TMP/SMX is associated with several significant adverse events that may limit pathogen-directed therapy, such as worsening renal function, hyperkalemia, and myelosuppression (23). Levofloxacin is less active against SM than either TMP/SMX or minocycline, and its role in treatment of minor infections is doubtful given the potential for the emergence of resistance during therapy (20).

The use of either minocycline or TMP/SMX in combination with one additional agent with activity against SM is the preferred approach to treat moderate to severe infections (20). Both of these agents demonstrated a high level of *in vitro* activity against SM, and minocycline was active against 90% of TMP/SMX-resistant isolates. Levofloxacin should only be used in combination with a second active agent (TMP/SMX or minocycline) to treat moderate to severe SM infections (20).

In conclusion, these results confirm previous reports regarding resistance to carbapenems in ACB isolates and the potentially useful activity of minocycline against CRAB and TMP/SMX-resistant SM. In addition to its activity against ACB and SM, minocycline is active against several of the ESKAPE pathogens (*Enterococcus faecium*, *Staphylococcus aureus*, *Klebsiella pneumoniae*, *A. baumannii, Pseudomonas aeruginosa*, and *Enterobacter* species) and remains a consideration for empiric therapy in seriously ill patients where the presence of multidrug-resistant bacteria may limit treatment options (20).

## MATERIALS AND METHODS

### Isolate collection

A total of 2,551 clinical isolates were selected from isolates recovered from documented infections in 35 U.S. medical centers from 2014 to 2021. Isolates were limited to one per patient per infection episode. The isolates selected for testing included 1,029 ACB and 1,522 SM. Isolates were from a variety of infection types, including pneumonia in PHP, BSI, SSSI, UTI, IAI, and other sites. Bacterial species were identified by the submitting laboratory and confirmed by JMI Laboratories using standard microbiology methods and matrix-assisted laser desorption-time of flight mass spectrometry (Bruker Daltonics, Bremen, Germany).

### Susceptibility testing

The broth microdilution (BMD) method for antimicrobial susceptibility testing was performed. Results were interpreted by following CLSI guidelines (24, 25). CLSI interpretive criteria for minocycline are: susceptible (S), ≤4 mg/L; intermediate (I), 8 mg/L; and resistant (R), ≥16 mg/L (25). Minocycline, levofloxacin, and meropenem were tested against isolates of ACB while minocycline, levofloxacin, and TMP/SMX were tested against SM. All antimicrobial agents were obtained from either US Pharmacopeia (North Bend, MD, USA) or Sigma-Aldrich (St. Louis, MO, USA).
